# Multidimensional toxicity of snake venom causes multi-organ damage and treatment challenges: a narrative review

**DOI:** 10.3389/fphar.2026.1815953

**Published:** 2026-05-29

**Authors:** Dan Tong, Long Chen, Jian Xu

**Affiliations:** 1 The Affiliated Hospital of Shaoxing University, Shaoxing, China; 2 School of Medical Technology and Information Engineering, Zhejiang Chinese Medical University, Hangzhou, China

**Keywords:** snakebite, toxic components, multi-organ damage, neurotoxic, hemotoxic

## Abstract

Snakebite envenomation has become a global public health challenge due to the widespread distribution of venomous snakes. Snake venom, a complex mixture containing various bioactive components, exhibits distinct characteristics across different families. The core toxic components of snake venom are mainly Three-Finger Toxins (3FTxs), phospholipase A2(PLA2), and proteases, which together form the material basis of the venom’s multidimensional toxicity. Through synergistic effects, they activate common pathological pathways, enabling targeted disruption of multiple human organs., leading to acute injury and even multi-organ failure. Beyond the acute effects, some survivors may experience long-term sequelae such as chronic kidney disease or permanent musculoskeletal damage. Existing research suggests that snake venom may have modulatory effects on the immune system, however, the relevant evidence primarily comes from *in vitro* experiments or animal models, and its clinical significance requires further validation. In clinical management, treatment for snake envenomation involves immediate wound care, prompt medical attention, and rapid diagnosis to identify the snake species for the timely administration of specific antivenom, and a multidisciplinary collaborative treatment model. Moreover, adjunctive drug therapy is often necessary. Nevertheless, traditional antivenoms still face challenges in addressing local tissue damage and ensuring accessibility in resource-limited regions. This narrative review focuses on three major toxin families of snake venom toxins, analyzing their molecular characteristics and synergistic mechanisms to elucidate how these toxins induce systemic damage affecting the cardiovascular, neurological, and renal systems. It thereby reveals that multi-organ injury caused by snake venom is not an isolated event but rather a systemic process driven by the interplay and synergy of multiple common pathological pathways. This systematic analysis suggests that clinical management should shift from a single-organ treatment approach to a systemic intervention strategy.

## 1 Introduction

Snakebite is a widespread and serious public health challenge globally, according to the WHO, between 4.5 and 5.4 million people are bitten by snakes each year. Of these, 1.8 to 2.7 million develop clinical illness, and 81,000 to 138,000 die from complications (World Health Organization, 2025), with severity ranging from asymptomatic bites to severe venom poisoning and even death. Research indicates that the incidence of severe systemic envenoming following snakebite is 14%, while the rates for severe tissue injury and severe hematologic toxicity are 14% and 18% (Gerardo et al., 2019).

The authoritative review (Gutiérrez et al., 2017) systematically summarizes the epidemiology, toxicological mechanisms, and clinical management of snakebite envenomation, laying a solid foundation for the field and representing a classic “toxin-by-toxin, system-by-system” discourse paradigm. However, although traditional reductionist approaches help identify individual toxin components, they fall short of capturing the systemic and complex pathological changes induced by snake venom, highlighting an urgent need for a more integrated and holistic research perspective (Gutiérrez et al., 2018). A fundamental question remains inadequately addressed: how do the multidimensional toxic components of snake venom interact synergistically at the molecular, cellular, and systemic levels to ultimately result in multiorgan damage? Answering this question is not only crucial for understanding the nature of the disease but also directly influences the selection of clinical treatment strategies.

The essence of snake venom-induced multiorgan damage lies in the synergistic action of multiple toxin families that simultaneously initiate mutually reinforcing pathological cascades across various physiological systems. Research has shown that snake venom toxins exhibit significant multifunctionality, with the same toxin family (e.g., phospholipase A2) capable of mediating multiple effects—such as pain, inflammation, and necrosis—through distinct structural domains (Ferraz et al., 2019), thereby providing a molecular basis for understanding synergistic interactions among toxins. This insight necessitates a shift in snake venom research from a linear thinking mode to a systematic approach integrating toxin biology, pathophysiology, and clinical medicine. Accordingly, this paper proposes an integrative conceptual framework termed the “Toxin–Pathway–Organ Axis,” which explicitly comprises three core components: (1) Input: three major toxin superfamilies (three-finger toxins, phospholipase A_2_, and proteases); (2) Intermediate processes: four shared pathological pathways (direct cytotoxicity, coagulation dysfunction, hemodynamic disturbances, and systemic inflammatory response); (3) Output: Specific organ damage involving the cardiovascular, nervous, and renal systems, as well as long-term sequelae. Compared with previous reviews, the novelty of this framework lies in its departure from the traditional approach of describing organ damage in isolation; instead, it defines multi-organ injury for the first time as a systemic process driven by shared pathological pathways with mutual reinforcement, thereby directly pointing to a fundamental shift in clinical treatment strategies.

The multidimensional toxicity of snake venom stems from compositional differences in venom composition among snakes of various families. Elapidae venoms are predominantly neurotoxic, Viperidae venoms are primarily hemotoxic (Table 1), while Colubridae venoms exhibit evolutionary diversity. Notably, the toxin spectra of these two families are not entirely discrete—certain phospholipases A2 in viper venoms can display neurotoxicity (Osip et al., 2023), whereas components of colubrid venoms may exhibit either elapid-like (neurotoxic) or viperid-like (hemotoxic) characteristics depending on the species (McGivern et al., 2014). This toxin spectrum feature, characterized by a clear foundational framework and mutually permeable boundaries, constitutes the core basis of the multidimensional snake venom toxicity and provides an entry point for understanding the complex clinical manifestations of simultaneous involvement of multiple systems (e.g., neurological, circulatory, and renal) following snakebite.

**TABLE 1:** Differences in venom of snake species in the families Elapidae and Viperidae.

Classification characteristic	Elapidae	Viperidae
Main types of toxins	Neurotoxins (e.g., 3FTxs, PLA2)	Hematologic toxicity (e.g., metalloproteinases, serine proteases)
Mechanism of toxic action	Blocks neuromuscular transmission, leading to paralysis and respiratory failure	Disrupts blood clotting function, causing bleeding, tissue necrosis, and blood vessel damage
Representative toxin	α-neurotoxins, short-chain neurotoxins	Metalloproteinases,thrombin-like enzyme
Secondary toxin	Small amounts of cytotoxin and cardiotoxin	Partial phospholipase A2
Clinical effect	Muscle paralysis, dyspnea, death (respiratory failure)	Local swelling, bleeding, shock, and renal failure
Representing snake species	Naja, bungarus, ophiophagus	Gloydius, crotalus, echis

Representative literature supporting the classification characteristics in this table can be found in: 3FTxs (Osipov et al., 2025), PLA2 (Nielsen et al., 2019; Šribar et al., 2019), α-neurotoxins (Khan et al., 2020), short-chain neurotoxins (Tan et al., 2019), metalloproteinases (Olaoba et al., 2020; Gutiérrez et al., 2016), serine proteases (Carone et al., 2018a; Megale et al., 2018).

Within this framework, this review focuses on the molecular characteristics and synergistic mechanisms of three major toxin families of snake venom (three-finger toxins, phospholipases A2, and proteases), and further elucidates how these toxins induce systemic damage to the cardiovascular, neurological, and renal systems through common pathways such as vascular injury, coagulation disorders, and inflammatory activation. On this basis, it also explores the limitations of current clinical treatment strategies and future directions.

## 2 Chemical composition and toxicological characteristics of the main toxins in snake venom

Snake venom is a complex mixture containing multiple bioactive components. Among the venoms of Elapidae, Viperidae, and Colubridae families, the core toxic constituents are primarily concentrated in three major superfamilies: three-finger toxins, phospholipases A2, and proteases. As systematically summarized by Ferraz et al., these toxin families not only exhibit multiple functions individually but also act synergistically to reinforce each other and collectively drive overall venom toxicity (Ferraz et al., 2019). This understanding is crucial for comprehending the mechanisms of multi-organ injury described in the subsequent sections.

### 2.1 Structure and function of three-finger toxins

**FIGURE 1:**
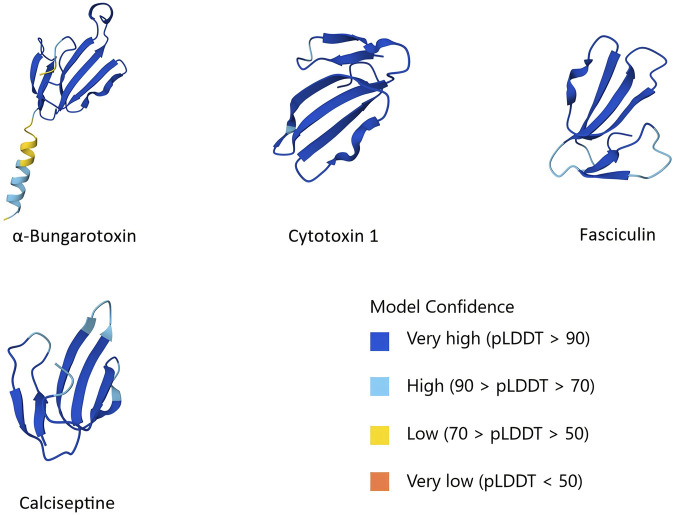
Structural diagram of common snake venom three-finger toxins. Source from: UniProt (https://www.uniprot.org).

3FTxs are a large family of snake venom peptides that share a similar structure but exhibit diverse functions. They are named for their core structural feature, the three-finger folding pattern, primarily found in Elapidae and certain Colubrid species. From a structural perspective, while sharing a core structure, they achieve distinct biological activities through subtle amino acid variations. This structural flexibility enables interactions with various biomolecules, resulting in diverse biological effects (Aoki-Shioi et al., 2020). 3FTxs exhibit different toxicities and biological functions across snake species (Nirthanan, 2020). From the perspective of mechanism of action and clinical relevance, among the traditional functions of 3FTxs, neurotoxicity is the most prominent. However, recent studies have shown that their functional diversity is far greater than previously anticipated (Table 2; Figure 1). In addition to neurotoxicity, certain members exhibit cytotoxicity, damaging cell membranes and causing tissue necrosis; others possess the ability to modulate enzyme activity, and some members can even act on smooth muscle. This implies that if the venom of a single snake species contains multiple types of 3FTxs, it can simultaneously trigger nerve, muscle, and local tissue damage, exemplifying the multidimensionality of snake venom at the toxin level.

**TABLE 2:** Structural and biological effects of different Three-Finger Toxins.

Toxicological characteristics	Protein name	Organism	Main mechanism of action	Clinical significance
Postsynaptic neurotoxin	α-Bungarotoxin	Bungarus multicinctus	Competitively blocks nAChR, causing neuromuscular paralysis	Respiratory failure, which can be neutralized by antivenom
Cytotoxicity	Cytotoxin 1	Naja melanoleuca	Disrupts the cell membrane, causing cell rupture and lysis	Local tissue necrosis, with limited neutralization by antivenom
Acetylcholine-sterase inhibitors	Fasciculin	Dendroaspis angusticeps	Inhibits AChE, disrupting neuromuscular impulse transmission	Muscle fasciculations, synergistically enhancing neurotoxicity
Mycotoxicity	Calciseptine	Dendroaspis polylepis	Relaxes smooth muscle and inhibits cardiac contraction	Cardiovascular dysfunction, exacerbation of shock

Representative literature supporting this table (Pruksaphon et al., 2020; Love and Stroud, 1986; Konshina et al., 2011; Konshina et al., 2010; le Du et al., 1996; Jadhav et al., 2013; de Weille et al., 1991). This table illustrates the correspondence between the multifunctionality of the same toxin family and its clinical effects.

The research evidence on 3FTxs is highly consistent in supporting their postsynaptic neurotoxic effects, which have been validated across multiple animal models and *in vitro* binding assays. Ferraz et al. further point out that through their conserved three-finger folding structure, 3FTxs can interact with a variety of receptors (nAChR, AChE, ion channels, *etc.*), thereby producing a diverse range of effects from neurotoxicity to cardiotoxicity (Ferraz et al., 2019). However, our understanding of their non-classical functions (such as cardiotoxicity) is primarily derived from biochemical experiments and *ex vivo* organ studies; the relative contribution of these effects to the envenoming process in humans, as well as their interactions with other toxins, remains speculative and requires further *in vivo* studies for validation.

### 2.2 Structure and function of phospholipase A2 toxin

**FIGURE 2:**
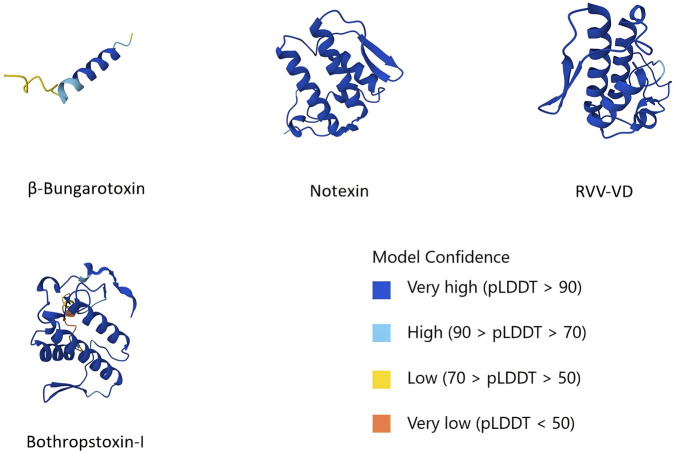
Structural diagram of common snake venom phospholipases A2. Source from: UniProt (https://www.uniprot.org).

From the perspective of core structural features, similar to 3-FTx, PLA2 in snake venom is also a diverse and numerous superfamily (Figure 2). Neurotoxicity has traditionally been the most prominent function of PLA2 (Bickler, 2020), but its functional diversity is equally remarkable (Table 3) and exhibits significant geographical variation. For example, in the venom of Bungarus multicinctus from China and its Taiwan Province, in addition to exhibiting strong neurotoxicity (Oh et al., 2021), PLA2 from certain snake venoms also possesses anticoagulant effects. Its anticoagulant activity facilitates the rapid spread of neurotoxic components (Kazandjian et al., 2021). Additionally, PLA2 may synergize with other toxins to enhance the overall toxicity of the venom. Research has identified that the synergistic action between PLA2 and 3FTx in the venom of the *Bungarus fasciatus* contributes to significant neurotoxicity and lethality (Hia et al., 2020). This synergistic effect suggests that the overall toxicity of snake venom is not a simple sum of the effects of individual toxins, but rather the result of synergistic and mutually reinforcing actions among different toxin families.

**TABLE 3:** Structural and biological effects of different phospholipases A2.

Toxicological characteristics	Protein name	Organism	Main mechanism of action	Clinical significance
Presynaptic neurotoxin	β-Bungarotoxin	Bungarus multicinctus	Inhibition of ACh release	Neuromuscular paralysis, with slow recovery (requiring newly synthesized synaptic vesicles)
Mycotoxicity	Notexin	Notechis scutatus	Enzymatic hydrolysis of the sarcolemma, calcium ion influx	Muscle necrosis, which may result in permanent functional disability
Anticoagulant toxicity	RVV-VD	*Daboia russelii*	Hydrolysis of the phospholipid platform in coagulation	Hemorrhagic tendency, which may exacerbate local and systemic bleeding
Muscle toxicity with antiplatelet activity	Bothropstoxin-I	Bothrops jararacussu	The modulation of platelet aggregation and function plays a key role in blood coagulation and thrombosis	Double injury: Hemostatic impairment and tissue necrosis

Representative literature supporting this table (Ivanušec et al., 2022; Halpert and Eaker, 1975; Nachtigall et al., 2022; Tsai et al., 1996; Suntravat et al., 2010; Ranéia et al., 2021a; Ranéia et al., 2021b). This table illustrates the correspondence between the multifunctionality of the same toxin family and its clinical effects.

The presynaptic neurotoxicity and direct myotoxicity mechanisms of PLA2 have been strongly supported in animal models. Ferraz et al. elaborated in detail on the multifunctionality of PLA2: the same PLA2 molecule can mediate myotoxicity via its C-terminal region, activate inflammatory responses and pain pathways through different structural domains, and even affect platelet function through its enzymatic or non-enzymatic mechanisms (Ferraz et al., 2019). However, there are inconsistent reports regarding the clinical relevance of its anticoagulant activity, which may be attributed to subtle structural differences in PLA2 from different snake species and variations in their plasma protein binding capacity. A critical knowledge gap is that, although *in vitro* experiments clearly demonstrate the diverse activities of PLA2, their temporal activation pattern during systemic envenoming and how they synergize with other toxins (particularly proteases) in inducing disseminated intravascular coagulation (DIC) are not yet fully elucidated.

### 2.3 Structure and function of protease toxins

**FIGURE 3:**
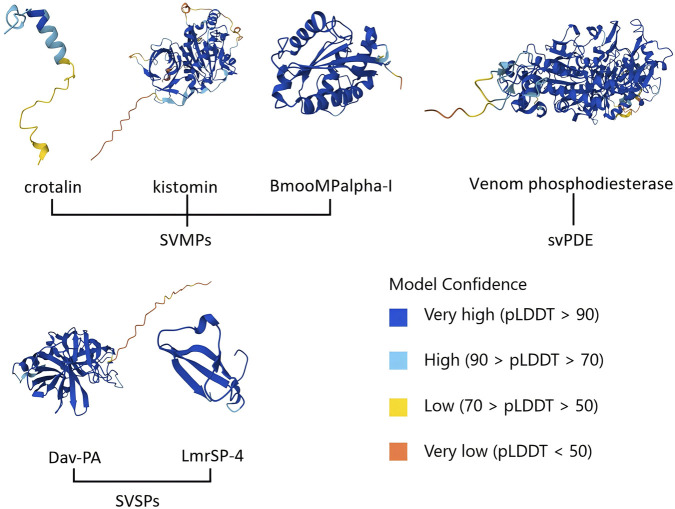
Structural diagram of common snake venom proteases. Source from: UniProt (https://www.uniprot.org).

Proteases constitute another core family of toxins in snake venom, comprising three major classes: snake venom metalloproteinases (SVMPs), snake venom serine proteases (SVSPs), and snake venom phosphodiesterases (svPDEs) (Figure 3). From a structural perspective, SVMPs exert their proteolytic activity by binding zinc ions (Nanjaraj Urs et al., 2015; Camacho et al., 2019), while SVSPs catalyze reactions through serine residues at their active sites (Carone et al., 2018b; Kang et al., 2011). Phosphodiesterases (svPDEs) are present in the venom of almost all snake families; although their content is low, they exert auxiliary toxicological effects. The clinical pathological damage caused by these toxins, particularly local tissue destruction and systemic hemorrhage following viperid snakebites, is closely associated with their action (Seneci et al., 2021; Alangode et al., 2020; Rudresha et al., 2020). Proteases mediate multi-organ damage through various pathways, snake venom metalloproteinases cause hemorrhage and tissue necrosis by degrading the vascular basement membrane, clinically presenting as local swelling, ecchymosis, and systemic bleeding, which can lead to shock or death in severe cases (de Souza et al., 2024); Snake venom serine proteases interfere with the coagulation cascade, leading to consumptive coagulopathy or disseminated intravascular coagulation, thereby exacerbating multi-organ damage (Sartim et al., 2017a); Phosphodiesterases participate in the pathological process by affecting the microcirculation (Table 4).

**TABLE 4:** Structural and biological effects of different snake venom proteases.

Type of protease	Active center/Coenzyme	Mainly distributed snake species	Primary toxicological action	Clinical significance
SVMPs	Zinc ion (Zn^2+^)	Viperidae, najaidae	Hemorrhage, tissue necrosis, impaired coagulation, induced inflammation	Primary mediators of local tissue necrosis and systemic hemorrhage; damage the vascular basement membrane, leading to systemic spread of toxins
SVSPs	Histidine, aspartic acid, serine	Viperidae, najaidae	Affecting coagulation (procoagulant/anticoagulant), lowering blood pressure, promoting fibrinolysis, and impacting platelet function	May cause consumptive coagulopathy or DIC; the effects are species-specific, making clinical prediction difficult
svPDE	Divalent metal ions	Venom found in almost all snake families	Regulate intercellular signal transduction and inhibit platelet aggregation	Low abundance but may amplify the effects of other toxins; long neglected and least studied

Representative literature supporting this table (Markland and Swenson, 2013; Oyama and Takahashi, 2017; Amel and Fatima, 2015; Du et al., 2025; Zelanis et al., 2015; Uzair et al., 2018). This table illustrates the correspondence between the multifunctionality of the same toxin family and its clinical effects.

As the primary mediators of hemorrhage and local tissue necrosis, the mechanisms of SVMPs have been consistently confirmed by extensive *in vivo* and *in vitro* studies. Gutiérrez et al. specifically emphasized that the degradation of microvascular basement membranes, disruption of endothelial cell–cell junctions, and subsequent inflammatory response induced by SVMPs together constitute a complex pathological network of local tissue damage, with many steps in this process—such as the DAMP-mediated inflammatory amplification—still urgently requiring elucidation (Gutiérrez et al., 2018). In contrast, the toxicological role of svPDE is currently the most insufficiently understood and speculative area in snake venom research. Future studies employing more refined approaches, such as gene knockout or specific inhibitors, are needed to clarify the true role of svPDE.

The diversity of snake venom components directly determines the complexity of its pathogenic mechanisms, different toxin families do not act in isolation, they collectively drive systemic toxicity through synergistic mechanisms. For example, PLA2 and 3FTxs can act on pre-synaptic and post-synaptic sites, respectively, achieving a dual blockade of neuromuscular transmission. The vascular wall destruction caused by SVMPs can accelerate the systemic spread of other toxin components, while the anticoagulant effect of PLA2 may exacerbate the bleeding tendency induced by SVMPs. It is precisely this multi-level, multi-target synergistic effect that ultimately leads to the multi-organ damage seen after snakebite envenoming: neurotoxins trigger respiratory failure, hemotoxins cause coagulation disorders and kidney injury, while tissue toxins result in local necrosis and systemic inflammatory responses.

However, several knowledge gaps remain in current research. Firstly, significant geographical and intraspecific variation exists in snake venom composition, the venom components and toxicity of the same snake species can differ markedly across different regions, posing a challenge for the precise formulation of antivenoms. Secondly, although current venomics techniques can identify hundreds of toxin components, the toxicological contribution of low-abundance toxins (e.g., svPDE) remains inadequately understood. Finally, most research has focused on the effects of individual toxins, while the dynamic synergistic processes and temporal relationships among different toxins *in vivo* require further elucidation. This complexity arising from toxin diversity, ultimately manifests in the specific multi-organ damage observed following snakebites.

## 3 Mechanism of multi-organ damage induced by snake venom

**FIGURE 4:**
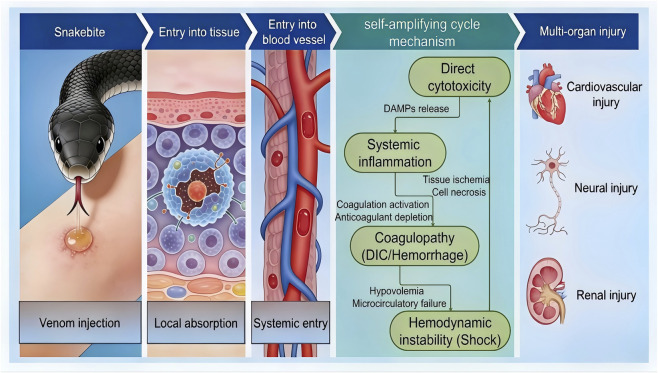
Diagram of Multi-Organ injury patterns induced by snake venom (By Figdraw).

When snake venom enters the human body, the toxic components rapidly disseminate through the bloodstream, triggering a complex and systemic pathophysiological process (Gamulin et al., 2025; Si et al., 2023). Based on the Toxin–Pathway–Organ Axis framework proposed in this paper, this chapter will elucidate how the three major toxin inputs lead to systemic damage in the cardiovascular, nervous, and renal systems through four shared pathological pathways (direct cytotoxicity, coagulation dysfunction, hemodynamic disturbances, and systemic inflammatory response) (Cavalcante et al., 2023; Sartim et al., 2025; Alvitigala et al., 2025) (Figure 4). These organ injuries are not independent events but are interconnected and mutually causative through common pathological mechanisms.

### 3.1 Damage mechanism of snake venom to cardiovascular system

The cardiovascular system is one of the primary targets of snake venom attack. The mechanisms of injury exhibit multi-site and multi-level complexity, including myocardial damage caused by cell membrane disruption, activation of coagulation pathways leading to hemostatic disorders, and vasodilation inducing blood stasis and even shock (Table 5). Differences in toxin combinations among various snake venoms result in clinical manifestations ranging from local edema to fatal circulatory failure. These toxins often accumulate and interact within the body to form a vicious cycle: hypotension induced by bleeding can exacerbate myocardial ischemia, while disseminated intravascular coagulation (DIC) further aggravates multi-organ hypoperfusion. Myocardial ischemia leads to heart failure, which in turn induces cardiogenic shock; heart failure worsens multi-organ hypoperfusion, further triggering hypotension and cardiogenic shock. This vicious pathological cycle rapidly progresses patients to circulatory failure in clinical settings.

**TABLE 5:** Mechanism of cardiovascular system damage and clinical manifestations of snake venom.

Types of damage	Main mechanism of action	Key toxin components	Pathophysiological changes and clinical manifestations
Direct myocardial injury	1. The toxins directly attack myocardial cells, damaging cell membranes, interfering with ion channels, inhibiting contractility, and inducing apoptosis 2. Cardiotoxins directly stimulate the myocardium and vascular smooth muscle	Cytotoxin, phosphatidase A_2_ Cardiac poison	Arrhythmia, heart failure, cardiogenic shock, elevated cardiac enzymes (cardiac troponin), early hypertension, followed by hypotension
Vascular damage and coagulation dysfunction	1. Destroys the vascular basement membrane, increases permeability, and induces hemorrhage 2. Excessive activation of the coagulation pathway consumes coagulation factors and triggers DIC 3. Inhibits coagulation factors or dissolves fibrin, exacerbating bleeding tendency	Metalloprotease Thrombin-like enzymes, coagulation factor X activators Proteolytic enzymes	Localized and systemic widespread bleeding, DIC, the blood is unable to clot
Vascular dysfunction	Induces the release of vasoactive substances such as histamine, bradykinin, and nitric oxide, leading to pronounced vasodilation and capillary leakage	Kallikrein, L-amino acid oxidase	Vasodilation, blood stasis, plasma extravasation, and reduced venous return result in refractory hypotension and distributed shock

Representative literature supporting this table: myocardial injury (Coimbra et al., 2024; Castro-Amorim et al., 2025; López-Dávila et al., 2021; Liblik et al., 2022); coagulation dysfunction (Escalante et al., 2011; Seo et al., 2017; Sartim et al., 2017b; Cheng et al., 2012; Isbister et al., 2015; Ogiwara et al., 2010); vascular dysfunction (Evangelista et al., 2011; Rucavado et al., 2018). This table is organized according to the structure of “injury type–mechanism–toxin–clinical manifestation,” reflecting the logical progression from input to output within the Toxin–Pathway–Organ Axis framework.

From the perspective of clinical manifestations, the aforementioned mechanisms collectively lead patients into a state of mixed shock: involving hypovolemic shock caused by hemorrhage/capillary leakage, distributive shock due to vasodilation, and potentially a cardiogenic shock component resulting from myocardial injury. This explains why circulatory failure following a snakebite progresses rapidly and is difficult to correct, while also highlighting the urgency of early administration of antivenom and concurrent circulatory support.

### 3.2 Toxicity effects of snake venom on the nervous system

**FIGURE 5:**
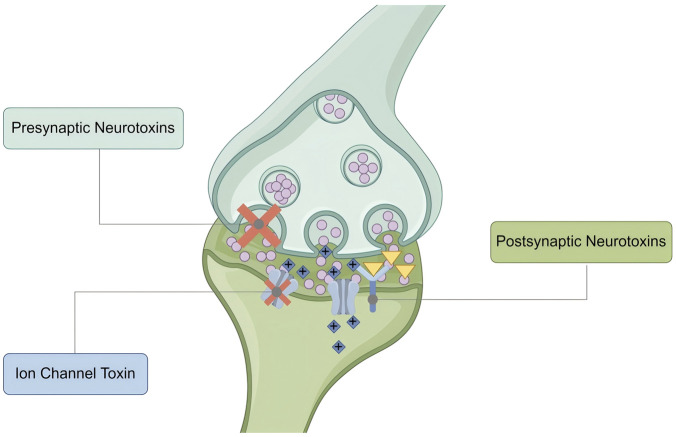
Schematic diagram of the mechanism of action of snake venom neurotoxin (By Figdraw).

The neurotoxins in snake venom can specifically target critical areas of the nervous system, disrupting the normal transmission of nerve signals, which can lead to a range of severe, even fatal symptoms, from muscle paralysis to respiratory failure. The core pathological basis of these symptoms lies in dysfunction at the neuromuscular junction, the key synapse through which neurons control muscle contraction and a common target for various neurotoxins. Based on their action sites, neurotoxins can be classified into three categories (Table 6; Figure 5), which exhibit significant differences in onset speed, reversibility, and clinical significance. This classification has important implications for guiding clinical treatment strategies.

**TABLE 6:** Mechanism of action and clinical significance of snake venom neurotoxin.

Parameter	Presynaptic neurotoxins	Postsynaptic neurotoxins	Ion channel toxins
Representative toxin	β-Bungarotoxin	α-Bungarotoxin	Dendrotoxin
Site of action	Neuronal terminal (presynaptic membrane)	nAChR receptors in muscle cells (postsynaptic membrane)	Voltage-gated ion channels on the axonal membrane of neurons
Primary mechanism	Possesses PLA2 activity and inhibit the release of acetylcholine (ACh)	Competitively and highly affinately binds to nAChR, blocking ACh binding	Toxins block or modulate ion channels, thereby affecting the generation and conduction of nerve impulses
Rate of onset	Relatively slow	Relatively fast	Usually very fast
Recovery time	Extremely slow (requires the formation of new synaptic vesicles)	May be relatively rapid (recover after toxin metabolism)	Variable (depending on channel type and function)
Clinical significance	Flaccid paralysis; onset is slow but recovery is extremely slow once it occurs, posing significant danger	Flaccid paralysis; rapid onset, recoverable after timely life support	Complex neurological dysfunctions (tremors, convulsions, *etc.*)

Representative literature supporting this table: Presynaptic neurotoxins (Prijatelj et al., 2008a; Davletov et al., 2012; Barber et al., 2012; Marcon and Nicholson, 2011; Prijatelj et al., 2008b; Silva et al., 2017); postsynaptic neurotoxins (Utkin, 2013; Tsetlin, 2015; Liang et al., 2020; Rosso et al., 2015; Huynh et al., 2022); ion channel toxins (Khan et al., 2018). This table clearly distinguishes the sites of action, mechanisms, and clinical significance of the three types of neurotoxins, reflecting the logical framework of input (toxin type)–intermediate process (mechanism of action)–output (clinical effect).

Knowledge regarding the mechanisms of neurotoxin action primarily derives from classical studies using isolated neuromuscular preparations (e.g., phrenic nerve-phrenic muscle). These models strongly demonstrate the existence of presynaptic and postsynaptic blockade. Existing research consistently emphasize that presynaptic neurotoxins (such as β-cyanoarachidonic acid neurotoxin), through their PLA2 domain, not only deplete synaptic vesicles but also enter the nerve terminal interior to attack mitochondria, thereby causing irreversible damage to nerve terminals—which explains why recovery from such envenoming is extremely slow (Gutiérrez et al., 2017; Ferraz et al., 2019). However, translating these mechanistic findings into precise temporal profiles of the human envenoming process still presents knowledge gaps, particularly regarding the dynamic synergistic ratios of different toxins *in vivo* and the mechanisms underlying individual variability in susceptibility to neurotoxins.

It is noteworthy that neurotoxins with different mechanisms of action often coexist synergistically within the same venom, significantly enhancing overall toxicity. For example, in Micrurus browni venom, the combination of PLA2 and three-finger toxins results in a marked increase in toxicity (Possani et al., 2020), similarly, presynaptic and postsynaptic neurotoxins in Dendroaspis venoms exhibit synergy, leading to complex neurotoxic symptoms (Jones et al., 2025). This synergistic interaction holds significant clinical implications: even with prompt administration of antivenom, nerve terminal damage caused by presynaptic toxins may result in delayed recovery of respiratory muscle function, necessitating continued respiratory support. From a clinical perspective, neurotoxic envenoming often begins with ophthalmoplegia (diplopia, ptosis), which can rapidly progress to dysphagia and cervical muscle weakness. In severe cases, it can affect the respiratory muscles, leading to respiratory failure—the speed of this progression varies by snake species. For instance, a bite from a Many-banded krait can be fatal within hours if untreated, underscoring the critical need for emergency intervention.

### 3.3 Effects of snake venom on renal function

**FIGURE 6:**
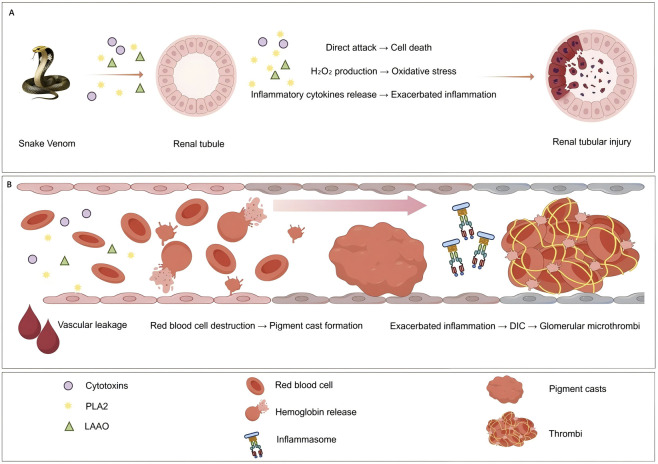
Schematic diagram of snake venom-induced kidney injury mechanism (By Figdraw). **(A)** Direct damage to the kidney by snake venom. **(B)** Indirect damage to the kidney by snake venom.

Kidney damage caused by snake venom is one of its most dangerous complications, often leading to acute kidney injury or even failure (Wijewickrama et al., 2020; Li et al., 2016). Epidemiological data indicate that the incidence of acute kidney injury ranges from 14.1% to 29.1% and is significantly associated with mortality (Pushpalatha et al., 2024; Moon et al., 2024). The mechanisms of injury can be classified into direct and indirect types, which often coexist and exacerbate each other (Figure 6). Direct mechanisms involve cytotoxins and PLA2 in the venom, which directly attack and kill renal tubular epithelial cells, leading to acute tubular necrosis (Chaisakul et al., 2021; Rao et al., 2025). Research demonstrates that PLA2 in snake venom not only exhibits cytotoxicity but also exacerbates kidney damage through oxidative stress and inflammatory cytokine release. For instance, Asp-49 and Lys-49 PLA2 from Bothrops pauloensis venom can significantly reduce the glomerular filtration rate (GFR) and increase oxidative stress and inflammatory factor levels in renal tissues. Additionally, Bothrops alternatus venom has been shown to induce cytokine expression and oxidative stress in renal cells, thereby altering renal function (Marinho et al., 2021). Beyond PLA2, other components in snake venom, such as L-amino acid oxidase (LAAO), also significantly impact renal function. LAAO generates hydrogen peroxide (H_2_O_2_), triggering oxidative stress and leading to cell death (Hiu and Yap, 2020; Costal-Oliveira et al., 2019). These mechanisms are primarily derived from animal model studies, although tubular necrosis has been confirmed in human autopsies.

Indirect renal effects of snake venom are more common than direct damage, reflecting the renal component of a systemic pathological process. First, snake venom-induced hemorrhage, vascular leakage, and shock cause a sharp decline in renal perfusion, the most common etiology of acute tubular necrosis (Albuquerque et al., 2020). Second, hemoglobin released from hemolytic destruction of erythrocytes and myoglobin from muscle injury mediated by myotoxins collectively obstruct and damage the renal tubules, with the formation of pigmented casts (Albuquerque et al., 2019). Furthermore, snake venom activates the complement system, generating anaphylatoxins that exacerbate the inflammatory response (Luchini et al., 2019). Meanwhile, disseminated intravascular coagulation (DIC) leads to the formation of microthrombi in the glomeruli, further aggravating renal ischemia (Teixeira et al., 2019).

From the perspective of clinical presentation and prognosis, early signs of kidney injury include reduced urine output and “cola-colored” urine. Without timely intervention, severe cases can rapidly progress to anuric acute renal failure. The key to treatment lies in a dual strategy: administering a sufficient dose of antivenom as early as possible, while actively providing fluid resuscitation and urine alkalization to protect the renal tubules (Lertsakulbunlue et al., 2024; Hashimoto et al., 2025; Fahey et al., 2024). Once the patient enters the stage of renal failure, hemodialysis is required to sustain life (Tchaou et al., 2020; Jayaraman et al., 2022). Notably, some patients may develop chronic kidney disease even after surviving the acute phase, necessitating long-term follow-up.

### 3.4 Common mechanisms underlying multi-organ damage and clinical integration

Although the damage caused by snake venom toxins to the cardiovascular system, nervous system, and kidneys exhibits distinct characteristics, several common pathological mechanisms persist throughout these processes. Following a snakebite, the degradation of the vascular basement membrane by certain venom components leads to endothelial injury and microvascular disruption. This not only forms the basis of hemorrhage in the cardiovascular system but also acts as the initiating factor for microvascular injury in the kidneys, while simultaneously potentially disrupting the blood-brain barrier and exacerbating nerve damage. Concurrently, some snake venoms can directly activate the complement system, releasing anaphylatoxins and recruiting inflammatory cells to infiltrate multiple organs, thereby amplifying tissue damage. Regarding the coagulation system, disseminated intravascular coagulation (DIC) not only affects the cardiovascular system but also contributes to multi-organ dysfunction by forming microthrombi that block the glomeruli and the small vessels supplying nerves. Furthermore, snake venom components induce cell death via free radical generation, with this oxidative stress-apoptosis mechanism being consistently demonstrated in cardiomyocytes, renal tubular epithelial cells, and neurons.

These mechanisms are interwoven and collectively explain why snakebites often manifest a systemic cascade of multi-organ failure: initial local tissue damage triggers systemic inflammation and coagulation activation, leading to shock and reduced renal perfusion, which in turn exacerbates organ ischemia, creating a vicious cycle. This demonstrates that multi-organ damage caused by snake venom is not an isolated events, but rather a systemic process driven by interconnected and synergistic mechanisms. This understanding necessitates a shift in clinical management from a single-organ treatment approach to a systemic intervention strategy—with antivenom as the cornerstone, supplemented by meticulous management including circulatory support, respiratory support, renal replacement therapy, and anti-inflammatory/anticoagulant measures. Any delay in implementing a single intervention may result in missing the critical window to interrupt this cascade reaction.

## 4 Long-term effects and neglected impacts of snake venom

The acute toxic effects of snake venom have long been widely recognized, but its long-term impact on the health of survivors is gradually gaining attention. After injection, snake venom does not simply metabolize and disappear from the body; the tissue damage and immune responses it triggers can lead to persistent health issues. Current evidence indicates that the long-term sequelae of snakebites are primarily concentrated in two main aspects: chronic kidney disease and permanent musculoskeletal damage (Ariga et al., 2021; Jayawardana et al., 2016). Hypotheses regarding broader immune system reprogramming or the risk of chronic inflammatory diseases still require further clinical research for confirmation (Babenko et al., 2020; Geron et al., 2017).

### 4.1 Long-term effects of snake venom on immune system

The long-term effects of snake venom on the immune system primarily stem from its potent initial immune response and potential tissue damage. The complex proteins and peptides in the venom act as antigens, stimulating the body to produce specific antibodies and memory immune cells. This may lead to faster and stronger immune responses (sometimes even dangerous allergic reactions) when encountering the same venom again in the future (Burin et al., 2018). More importantly, in severe poisoning cases, the direct tissue necrosis, hemorrhage, or organ damage (such as acute kidney injury) caused by the venom can initiate chronic inflammation or fibrosis during the repair process, which may impair organ function in the long term (Rao et al., 2024). Additionally, studies have shown that certain snake venom components may possess immunomodulatory properties (Wang and Qin, 2018), theoretically potentially influencing susceptibility to autoimmune diseases. However, long-term clinical evidence in this area is still being explored and is not yet a universal conclusion. Therefore, the extent of long-term effects largely depends on the severity of the poisoning, the snake species involved, the timeliness and efficacy of treatment, as well as the individual’s underlying health condition.

### 4.2 Chronic disease risk from snake venom

The risk of chronic diseases following snake venom poisoning primarily stems from the severe initial tissue damage and subsequent irreversible pathological changes caused by the venom. For instance, myotoxins or cytotoxins in the venom may lead to extensive muscle necrosis. Even after the wound healing, permanent muscle atrophy, fibrosis, and functional loss may persist, potentially resulting in disability in severe cases (López-Dávila et al., 2024; Yong et al., 2023). Kidney damage is particularly critical, as acute kidney injury can progress to chronic kidney disease. In a prospective study on acute kidney injury caused by snake venom, researchers found that although some patients recovered well during hospitalization, approximately 28.7% still experienced adverse long-term renal outcomes during follow-up, such as chronic kidney disease, hypertension, or even the need for long-term dialysis (Priyamvada et al., 2019). This data clearly indicates that acute kidney injury is a significant risk factor for chronic kidney disease, necessitating long-term monitoring. Furthermore, the anticoagulant components in the venom may cause widespread intravascular coagulation or hemorrhage, potentially damaging vascular endothelial cells and increasing the risk of future thrombosis. Extensive necrosis of local tissues often leads to long-term functional impairment, chronic pain, or sensory abnormalities. The risk of these chronic sequelae is directly related to the severity of the poisoning and the timeliness of treatment, underscoring the critical importance of prompt and effective medical intervention.

## 5 Treatment strategies for snake venom poisoning

The treatment of snakebite envenoming faces multiple challenges, including both the inherent limitations of existing therapies and issues of accessibility in resource-limited settings. Clinical management requires coordinated efforts across multiple aspects, including prehospital first aid, antivenom administration, and management of complications.

### 5.1 Standard treatment protocol

In emergency care, timely wound management and prompt medical attention are critical. For snakebite victims, strenuous activities should be avoided, and the wound should be kept below heart level to slow the venom spread (Hifumi et al., 2015; Di Nicola et al., 2021). In the diagnosis and treatment of snake envenomation, the use of antivenom remains the only effective therapeutic approach. Emphasis is placed on early, adequate, and intravenous administration, with the prerequisite for success being rapid diagnosis and identification of the snake species to select the corresponding serum (Williams et al., 2019; Minghui et al., 2019).

### 5.2 Limitations of current therapies

The clinical application of antivenom serum faces multiple limitations. Firstly, its efficacy is significantly influenced by geographical and species variations, with considerable differences in cross-reactivity among snake species. Inaccurate identification of the snake species can lead to suboptimal therapeutic outcomes (Xie et al., 2020; Kumkate et al., 2020). Secondly, while antivenom is effective in neutralizing circulating toxins, antivenom serum shows limited efficacy against established local tissue necrosis and hemorrhage, potentially leaving patients with permanent functional impairments (Liu et al., 2020). Thirdly, regions with high incidence, such as sub-Saharan Africa and South Asia, have long faced challenges such as insufficient antivenom supply, exorbitant costs, and storage difficulties. Fourthly, traditional animal-derived antivenom may trigger acute allergic reactions or serum sickness, restricting their clinical application.

### 5.3 Emerging therapeutic strategies

In response to the aforementioned limitations, and based on the systemic intervention strategy concept proposed in this review, we suggest two priority directions for emerging therapeutic strategies. First, develop broad-spectrum toxin inhibitors and adjunctive therapies targeting common pathways. Broad-spectrum inhibitors (e.g., small molecules or recombinant antibodies simultaneously targeting 3FTxs, PLA2, and SVMPs) are expected to address geographical and intraspecific variations in snake venom components, enhancing therapeutic universality. Adjunctive therapies targeting common pathways, such as anti-inflammatory drugs and antioxidants, can further block the mutually reinforcing pathological cycle leading to multiple organ failure after antivenom neutralization of the toxins. Second, establish a multidisciplinary collaborative treatment model. The systemic nature of snake venom injuries necessitates clinical management that transcends traditional single-organ treatment approach, shifting toward a comprehensive framework integrating intensive care (circulatory/respiratory support), renal support (renal replacement therapy), and rehabilitation medicine (management of long-term sequelae).

## 6 Perspectives

Snakebite envenomation is far more than a simple acute toxic event. This review clarifies that it constitutes a dynamic process involving complex chemical components, interactions across multiple organ systems in pathophysiology, and the potentially profound health consequences. Currently, research and clinical practice in this field are undergoing a critical paradigm shift: transitioning from the past focus on reducing immediate mortality towards the more comprehensive goal of comprehensively improving long-term patient outcomes and quality of life. The intrinsic logic of this transformation is precisely a profound response to the systemic nature of multi-organ damage induced by snake venom—therefore, clinical management must shift from a single-organ treatment approach to a systemic intervention strategy.

To achieve this goal, future research should focus on the following directions: Firstly, promoting innovation and breakthroughs in therapeutic strategies. Although antivenom remains the core means of neutralizing toxins, its effectiveness in mitigating local tissue damage and long-term sequelae is limited. Therefore, there is an urgent need to develop novel adjunctive drugs with enhanced targeting capabilities. Through the synergistic application of precision medicine and traditional antivenom therapy, a multi-modal, full-course intervention system should be established. Secondly, establishing a systematic framework for long-term health management. This review highlights long-term complications such as chronic kidney disease, permanent musculoskeletal damage, and the potential risk of prolonged immune system dysregulation, necessitate. By establishing a full-cycle health management model that spans from acute-phase treatment to long-term rehabilitation, we can not only to improve the survival rates of envenomed patients but also to significantly enhance their long-term quality of life and health outcomes. This approach will fundamentally alleviate the overall burden of this global public health challenge.
